# Energy harvesting thermocell with use of phase transition

**DOI:** 10.1038/s41598-020-58695-z

**Published:** 2020-02-04

**Authors:** Takayuki Shibata, Hiroki Iwaizumi, Yuya Fukuzumi, Yutaka Moritomo

**Affiliations:** 1 National Institute of Technology, Gunma College, Maebashi, Gunma 371-8530 Japan; 2 Graduate School of Pure and Applied Sciences, University of Tsukuba, Tsukuba, Ibaraki 305-8571 Japan; 3 Faculty of Pure and Applied Sciences, University of Tsukuba, Tsukuba, Ibaraki 305-8571 Japan; 4 Tsukuba Research Center for Energy Materials Science (TREMS), University of Tsukuba, Tsukuba, Ibaraki 305-8571 Japan

**Keywords:** Devices for energy harvesting, Phase transitions and critical phenomena, Batteries

## Abstract

A thermocell that consists of cathode and anode materials with different temperature coefficients (*α* = d*V*/d*T*) of the redox potential (*V*) can convert environmental thermal energy to electric energy via the so-called thermal charging effect. The output voltage *V*_cell_ of the current thermocell, however, is still low (several tens mV) and depends on temperature, which are serious drawbacks for practical use of the device as an independent power supply. Here, we report that usage of phase transition material as electrode qualitatively improve the device performance. We set the critical temperature (*T*_c_) for the phase transition in cobalt Prussian blue analogue (Co-PBA; Na_*x*_Co[Fe(CN)_6_]_*y*_) to just above room temperature, by finely adjusting the Fe concentration (*y* = 0.82). With increase in the cell temperature (*T*_cell_), *V*_cell_ of the Na_*x*_Co[Fe(CN)_6_]_0.82_ (NCF82)/Na_*x*_Co[Fe(CN)_6_]_0.9_ (NCF90) cell steeply increases from 0 mV to ~120 mV around 320 K. Our observation indicates that the thermocell with use of phase transition is a promising energy harvesting device.

## Introduction

A new energy harvesting technology from environmental heat, e.g., day and night temperature change, waste heat near room temperature, and human body heat, is required to realize the “smart” society. A semiconductor-based thermoelectric device that uses the so-called Seebeck effect is a promising technology and is practically used in Peltier cooling and thermal power generation in space vehicles^1^. Another energy harvesting technology with low cost and high efficiency is the thermocell that consists of cathode and anode materials with different thermal coefficients (*α* = d*V*/d*T*) of the redox potential (*V*) between the anode (*α*_anode_) and cathode (*α*_cathode_) materials. Several researchers^2–8^ reported that such a thermocell can convert environmental thermal energy to electric energy via the so-called thermal charging effect. Hereafter, we call such devices as “tertiary batteries”, because the cell can be charged by thermal energy not by electric energy. Strictly speaking, the battery converts thermal energy to electric energy in a thermal cycle between low (*T*_L_) and high (*T*_H_) temperatures. This makes in sharp contract with the semiconductor-based thermoelectric device that converts the permanent temperature gradient to electric energy. In the warming process, the battery shows a cell voltage (*V*_cell_) of (*α*_cathode_−*α*_anode_) (*T*_H_ − *T*_L_). The thermally-charged energy is converted to the electric energy in the discharge process at *T*_H_. Similarly, the cooling process induces *V*_cell_ [= − (*α*_cathode_−*α*_anode_) (*T*_H_ − *T*_L_)]. The thermally-charged energy is converted to the electric energy in the discharge process at *T*_L_. Fukuzumi *et al*.^6^ fabricated a thermocell, consisting of two types of PBA films, Na_*x*_Mn[Fe(CN)_6_]_0.83_ (MCF83) and Na_*x*_Co[Fe(CN)_6_]_0.9_ (NCF90), with different *α* values. The NMF83/NCF90 cell shows a thermal voltage of *V*_cell_ ~ 40 mV in the thermal cycle between *T*_L_ (=286 K) and *T*_H_ (=313 K). The tertiary battery extends the application range of the battery materials from energy storage to energy harvesting (or independent power supply).

The output voltage *V*_cell_ [= (*α*_cathode_−*α*_anode_) (*T*_H_ − *T*_L_)] of the conventional thermocell has two drawbacks for practical use as an independent power supply for information technology (IT) and/or internet of things (IoT) devices. One is that the current *V*_cell_ (several tens mV) is too low to drive the IT/IoT devices. For example, the NMF83/NCF90 cell (*α*_cathode_−*α*_anode_ = 1.7 mV/K)^6^ shows a thermal voltage of *V*_cell_ ~ 40 mV between *T*_L_ (=286 K) and *T*_H_ (=313 K). So far, *α* of wide range of battery materials were reported; *α* = −0.3–1.4 mV/K in Prussian blue analogues^8^, *α* = 0.2–1.1 mV/K in several conjugated polymers^9^, *α* = 0.0−0.9 mV/K in Na_*x*_CoO_2_^10^ and *α* = 0.9 mV/K in Li_*x*_FePO_4_^11^. The reported |*α*| is order of 1 mV/K at the maximum, indicating that a more elaborated exploration is indispensable to obtain much higher-*V*_cell._ of order of several hundred mV. Another drawback is that *V*_cell_ is not constant but is proportional to Δ*T* (=*T*_H_ − *T*_L_). Such a temperature dependence (*T*-dependence) of *V*_cell_ is unsuitable as power supply for the IT/IoT devices.

**Figure 1:**
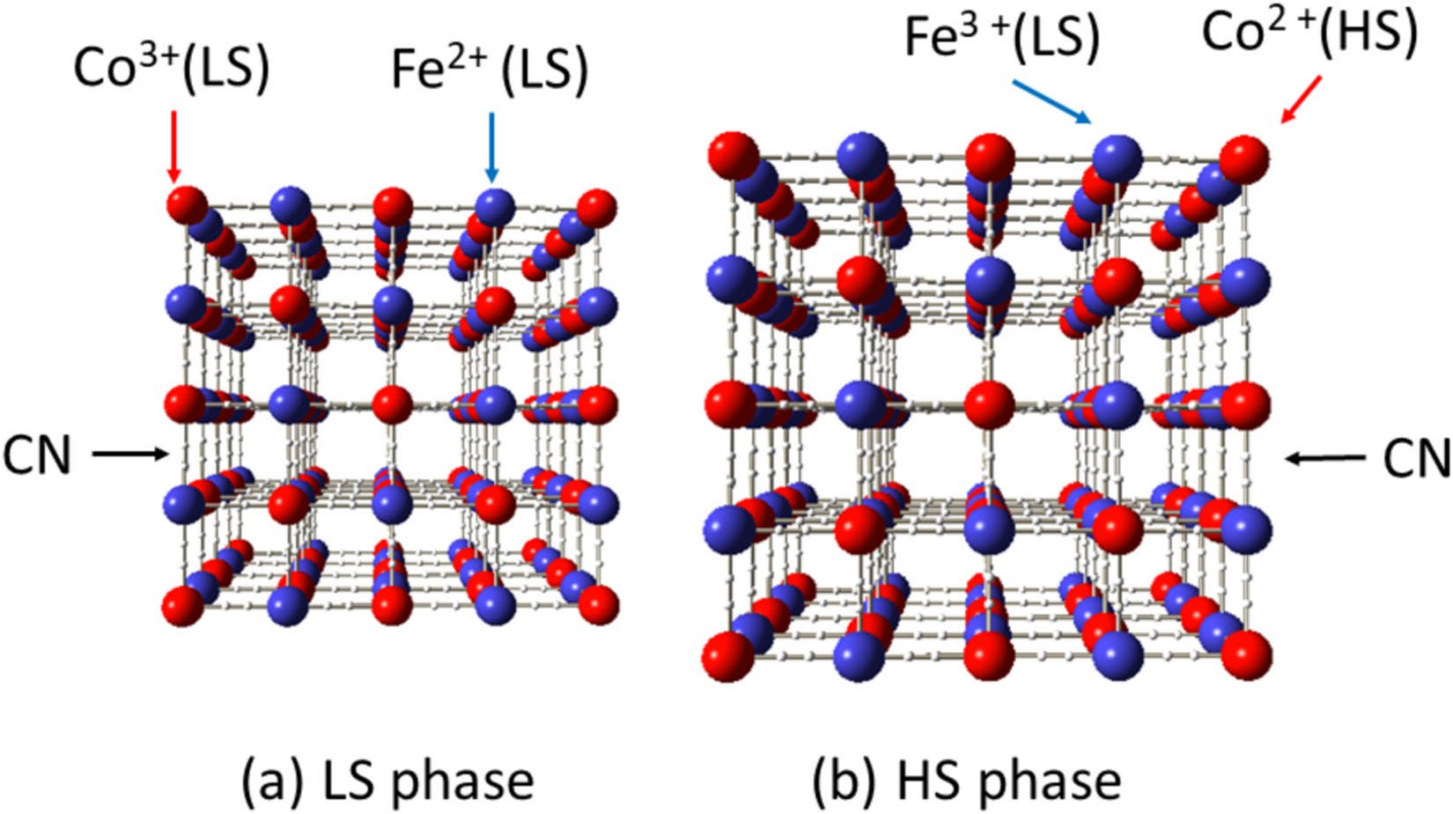
Schematic structure of Na_x_Co[Fe(CN)_6_]_y_ (Co-PBA) in the (**a**) low-spin (LS) and (**b**) high-spin (HS) phases. For simplicity, guest ions (Na^+^) are omitted. The LS–HS phase transition is triggered by cooperative charge transfer from Fe^2+^ to Co^3+^, which causes spin state transition of Co from LS Co^3+^ to HS Co^2+^.

The usage of phase transition material as electrode is expected to solve the above-mentioned two drawbacks, i.e., low-*V*_cell_ and *T*-dependence of *V*_cell_. The structural phase transition discontinuously changes the electronic and electrochemical material parameters via variation of the crystal structure (including valence state and spin state of the constituent elements). If a phase transition material is used as electrode, *V*_cell_ is expected to change significantly when *T*_cell_ crosses *T*_c_. In addition, if the variation of *V*_cell_ at *T*_c_ overwhelms the conventional temperature dependence of *V*_cell_ [= (*α*_cathode_ − *α*_anode_) (*T*_H_ − *T*_L_)], we can neglect the latter effect. The cobalt Prussian blue analogue (Co-PBA; Na_*x*_Co[Fe(CN)_6_]_*y*_) is a promising cathode material for Li^+^/Na^+^ secondary battery^12–14^. Co-PBA have face-centered cubic (fcc) (*Fm*$\bar{3}$*m*; *Z* = 4) or trigonal (*R*$\bar{3}$*m*; *Z* = 3) structures^15^, consisting of a three-dimensional (3D) jungle-gym-type host framework with guest Li^+^/Na^+^ ions. Figure 1 shows schematic structure of Co-PBA.

Importantly, Co-PBA shows a characteristic first-order phase transition from low-spin [LS: Fig. 1(a)] phase to high-spin [HS: Fig. 1(b)] phase. The LS– HS phase transition is triggered by the cooperative charge transfer from Fe^2+^ to Co^3+^^16^. The electronic configuration changes from Co^3+^ − Fe^2+^ (LS phase) to Co^2+^ − Fe^3+^ (HS phase). The resultant valence change in Co changes the electronic configuration of Co from LS Co^3+^ to HS Co^2+^^17^ and increases the ionic radius of Co from 0.65 Å (LS Co^3+^) to 0.75 Å (HS Co^2+^). By contrast, Fe takes the LS configuration in both the divalent and trivalent states. The ionic radii of Fe are almost the same, 0.61 Å (LS Fe^2+^) to 0.55 Å (LS Fe^3+^). The phase transition accompanies significant increase in the cell volume, reflecting the larger ionic radius (0.75 Å) of HS Co^2+^. Therefore, we can expect a significant variation of the redox potential (*V*) at the LS – HS phase transition in Co-PBA.

Here, we demonstrated that the usage of the LS–HS phase transition of Co-PBA (Na_*x*_Co[Fe(CN)_6_]_*y*_) qualitatively improved the device performance. We set *T*_c_ of the LS–HS transition to just above room temperature, by finely adjusting the Fe concentration (*y* = 0.82). With increase in *T*_cell_, *V*_cell_ of the Na_*x*_Co[Fe(CN)_6_]_0.82_ (NCF82)/Na_*x*_Co[Fe(CN)_6_]_0.9_ (NCF90) cell steeply increase from 0 mV to ~ 120 mV around 320 K. Our observation indicates that the tertiary battery with use of phase transition is a promising independent power supply for the IT/IoT devices.

## Phase Diagram of NCF82

The critical temperature of the LS–HS transition of Na_*x*_Co[Fe(CN)_6_]_*y*_ can be finely controlled by the concentration (*y*) of Fe(CN)_6_^16^, which octahedrally coordinates the Co site. With increase in *y*, the ligand field at the Co site becomes stronger to stabilize the trivalent LS Co state. In other words, *T*_c_ of the LS–HS phase transition increases with increase in *y*. Actually, Shimamoto *et al*.^16^ reported that the upper (*T*_c_^u^) and lower (*T*_c_^l^) critical temperature steeply increases with increases in *y*. At *y* = 0.87, no phase transition takes place below 350 K. Therefore, *T*_c_^u^ (*T*_c_^l^) of NCF90 (*y* = 0.90) is considered to be much higher than 350 K if exists. We note that nature of the phase transition depends not only on *y*, but also on the Na concentration (*x*). For example, in Na_*x*_Co[Fe(CN)_6_]_0.71_ (*y* = 0.71)^18^, nature of the phase transition changes from the first- to second-order type with decrease in *x*. By means of the magnetic susceptibility measurement, we systematically investigated *T*_c_^u^ and *T*_c_^l^ against y [Fig. S1(a,b)]. We found that *T*_c_^u^ and *T*_c_^l^ linearly increases with *y* and approaches to room temperature at *y* ~ 0.81. We finally concluded that Na_*x*_Co[Fe(CN)_6_]_0.82_ (NCF82) is the best composition for our experiment.

The LS–HS phase transition is easily detected by steep increase in the lattice constant (*a*). To determine the phase diagram of NCF82 against Na concentration (*x*), we systematically investigated *T*-dependence of *a* at various *x*. Figure S2 shows overall X-ray diffraction (XRD) pattern of NCF82 film against *x* at 300 K. The NCF82 shows the fcc (*Fm*$\bar{3}$*m*; *Z* = 4) structure below *x* = 0.96, even though the as-grown film shows trigonal (*R*$\bar{3}$*m*; *Z* = 3) structure. We note that Co-PBA will deteriorate if left at high temperature (>350 K) for a long time. To shorten the measurement time, only the (002) reflection in the cubic cell was investigated (Fig. S3). *a* was calculated with use of Bragg’s law.

**Figure 2:**
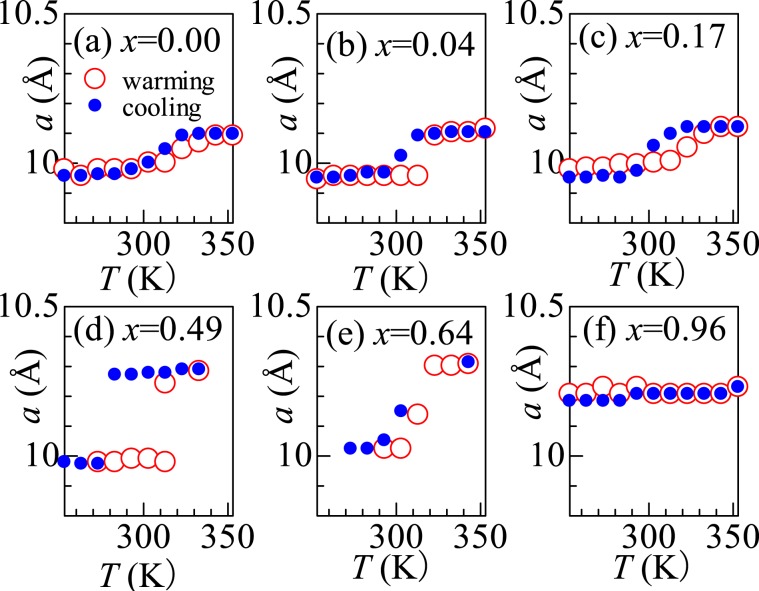
Temperature dependence of the lattice constant (*a*) of Na_*x*_Co[Fe(CN)_6_]_0.82_; (**a**) *x* = 0.00, (**b**) 0.04, (**c**) 0.17, (**d**) 0.49, (**e**) 0.64, and (**f**) 0.96. Open and closed circles represent the data obtained in the warming and cooling runs, respectively.

Figure 2 shows *T-*dependence of *a* of the NCF82 film at various *x*. The magnitude of *x* was evaluated from the extracted charge under the assumption that Na_1.28_Co[Fe(CN)_6_]_0.82_ is in the discharged state and Na_0.00_Co[Fe(CN)_6_]_0.82_ is in the fully-charged state. Open and closed circles represent the data obtained in the warming and cooling runs, respectively. At (a) *x* = 0.00, *a* gradually increases around 320 K in the warming run, indicating the phase transition from the LS phase to the HS phase. In the cooling run, *a* gradually decreases around 320 K, indicating the phase transition from the HS phase to the LS phase. *T*_c_^u^ (*T*_c_^l^) are defined by the temperature corresponding to the midpoint of *a* between the high temperature and low temperature sides in the warming (cooling) run. A similar increase (decrease) in *a* is observed at (b) *x* = 0.04 and (c) 0.17 in the warming (cooling) runs. At (d) *x* = 0.49, *a* discontinuously increases at *T*_c_^u^ = 315 K while it discontinuously decreases at *T*_c_^l^ = 280 K. The larger thermal hysteresis (=35 K) is perhaps ascribed to the significant variation (=0.3 Å) in *a*. A similar increase (decrease) in *a* is observed at (e) *x* = 0.64 in the warming (cooling) runs. *a* becomes essentially temperature-independent at (f) *x* = 0.96, indicating that that no phase transition takes place.

**Figure 3:**
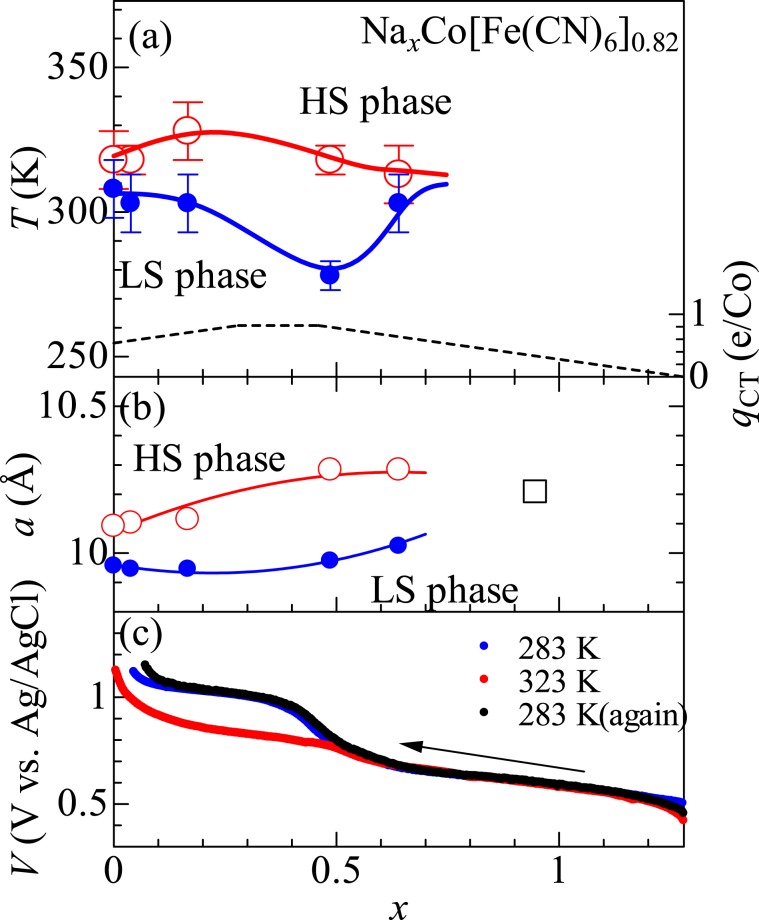
(**a**) Phase diagram of Na_*x*_Co[Fe(CN)_6_]_0.82_ against *x*. Open and closed circles represent upper (*T*_c_^u^) and lower (*T*_c_^l^) critical temperatures, respectively. The red and blue curves are just guide to the eyes. The broken curve represents calculated charge transfer (*q*_CT_) from Fe^2+^ to Co^3+^ at the LS–HS transition. (**b**) Lattice constant (*a*) against *x*, Open and closed circles represent *a* in the HS and LS phases, respectively. The red and blue curves are just guide to the eyes. Squares are the value at room temperature. (**c**) Charge curves of the NCF82 film at various temperatures. The charge rate was 0.6 C.

Figure 3(a) shows the *x* − *T* phase diagram. Open and closed circles represent *T*_c_^u^ and *T*_c_^l^, respectively. The red and blue curves just guide to the eyes. The HS phase is stable above the red boundary while the LS phase is stable below the blue boundary. In the region sandwiched by the red and blue boundaries, either phase appears depending on the thermal history. The width (Δ_c_ = *T*_c_^u^ − *T*_c_^l^) of the thermal hysteresis gradually increases with *x* from 15 K at *x* = 0.00 to 45 K at 0.49. With further increases in *x*, Δ*T*_*c*_ steeply decreases to 10 K at *x* = 0.64 and the phase transition disappears above *x* = 0.96. Figure 3(b) shows *a* in the HS (open circles) and LS (closed circles) phases against *x*. With increase in *x*, variation (Δ*a*) of *a* between the HS and LS phases gradually increases from Δ*a* = 0.15 Å at *x* = 0.00 to 3.5 Å at 0.49. With further increases in *x*, Δ*a* decrease to 0.25 Å at *x* = 0.64. Thus, Δ*T*_*c*_ correlates with Δ*a* at the LS–HS transition.

Here, let us consider the magnitude of the change transfer (*q*_CT_) from Fe to Co at the LS–HS transition. The phase transition is expressed as follows; Na_*x*_Co^III^[Fe^II^(CN)_6_]_0.54+*x*_[Fe^III^(CN)_6_]_0.28−*x*_ ➔ Na_*x*_Co^II^_0.54+*x*_Co^III^_0.46−*x*_[Fe^III^(CN)_6_]_0.82_. (*x* < 0.28), Na_*x*_Co^II^_*x*−0.28_Co^III^_1.28−*x*_[Fe^II^(CN)_6_]_0.82_ ➔ Na_*x*_Co^II^_0.54+*x*_Co^III^_0.46−*x*_[Fe^III^(CN)_6_]_0.82_ (0.28 < *x* < 0.46), and Na_*x*_Co^II^_*x*−0.28_Co^III^_1.28−*x*_[Fe^II^(CN)_6_]_0.82_ ➔ Na_*x*_Co^II^[Fe^II^(CN)_6_]_*x*−0.46_[Fe^III^(CN)_6_]_1.28−*x*_ (0.46 < *x*). The broken curve in Fig. 3(a) is the calculated *q*_CT_ against *x*. With increase in *x*, *q*_CT_ linearly increases from *q*_CT_ = 0.54 e/Co at *x* = 0.00 to 0.82 e/Co at 0.28 and becomes constant (= 0.82). With further increase in *x* beyond *x* = 0.46, *q*_CT_ linearly decrease from *q*_CT_ = 0.82 e/Co at *x* =  0.46 to 0.00 e/Co at 1.28. These *x*-dependent behavior of *q*_CT_ positively correlates with Δ*T*_*c*_ and Δ*a*. We note that the no phase transition is observed in the *x* region where *q*_CT_ < 0.5 e/Co.

## Effect of the Phase Transition on Redox Potential

The LS–HS phase transition has significant effect on the redox potential (*V*). Figure 3(c) shows charge curves of the NCF82 film at various temperatures. At *T* = 283 K in the LS phase, the curve shows two plateaus at 1.0 and 0.6 V vs. Ag/AgCl. By means of the X-ray absorption spectroscopy (XAS), Takachi *et al*.^13^ indicated that the lower- and higher-lying plateaus of NCF90, which is in the LS phase at 300 K are ascribed to the redox reaction of Fe and Co, respectively. Then, the higher-lying plateau of NCF82 in the LS phase is reasonably ascribed to Co^III^[Fe^III^(CN)_6_]_0.6_[Fe^II^(CN)_6_]_0.3_ + 0.6Na^+^  + 0.6 e^−^ ➔ Na_0.6_Co^III^[Fe^II^(CN)_6_]_0.9_ while the lower-lying plateau is ascribed to Na_0.6_Co^III^[Fe^II^(CN)_6_]_0.9_ + Na^+^  + e^−^ ➔ Na_1.6_Co^II^[Fe^II^(CN)_6_]_0.9_. At 323 K in the HS phase, the curve significantly changes. Especially, *V* in the higher-lying plateau significantly drops by about 150 mV. The potential drop cannot be ascribed to the sample deterioration, because the charge curve returns to the original value at 283 K. In the HS phase, the redox site of the higher-lying plateau is considered to be Co, because the valence state in the HS phase is Na_*x*_Co^II^_0.54+*x*_Co^III^_0.46−*x*_[Fe^III^(CN)_6_]_0.82_. (*x* < 0.28) or Na_*x*_Co^II^_0.54+*x*_Co^III^_0.46−*x*_[Fe^III^(CN)_6_]_0.82_ (0.28 < *x* < 0.46). Such a switching of the redox site causes the potential drop as observed.

This hypothesis, i.e., the redox site switching, is supported by the vibrational spectroscopy in the infrared (IR) region (Fig. S4). In the LS phase (298 K), the CN stretching vibrational mode is observed around 2080–2020 cm^−1^ in the lower-lying plateau while an additional band appears at 2200 cm^−1^ in the higher-lying plateau. The former (latter) bands ascribed to the CN stretching vibrational mode in the [Fe(CN)_6_]^4−^ ([Fe(CN)_6_]^3−^) units. Therefore, in the LS phase, the redox cites in the lower- and higher-lying plateaus are Co and Fe, respectively. In the HS phase (330 K), the corresponding mode is observed around 2240–2200 cm^−1^ in the higher-lying plateau while an additional band appears at 2080 cm^−1^ in the lower-lying plateau. Accordingly, in the HS phase, the redox cites in the lower- and higher-lying plateaus are Fe and Co, respectively. Thus, the IR spectroscopy indicates the redox site switching at the LS–HS phase transition.

## NCF82/NCF90 Tertiary Battery

**Figure 4:**
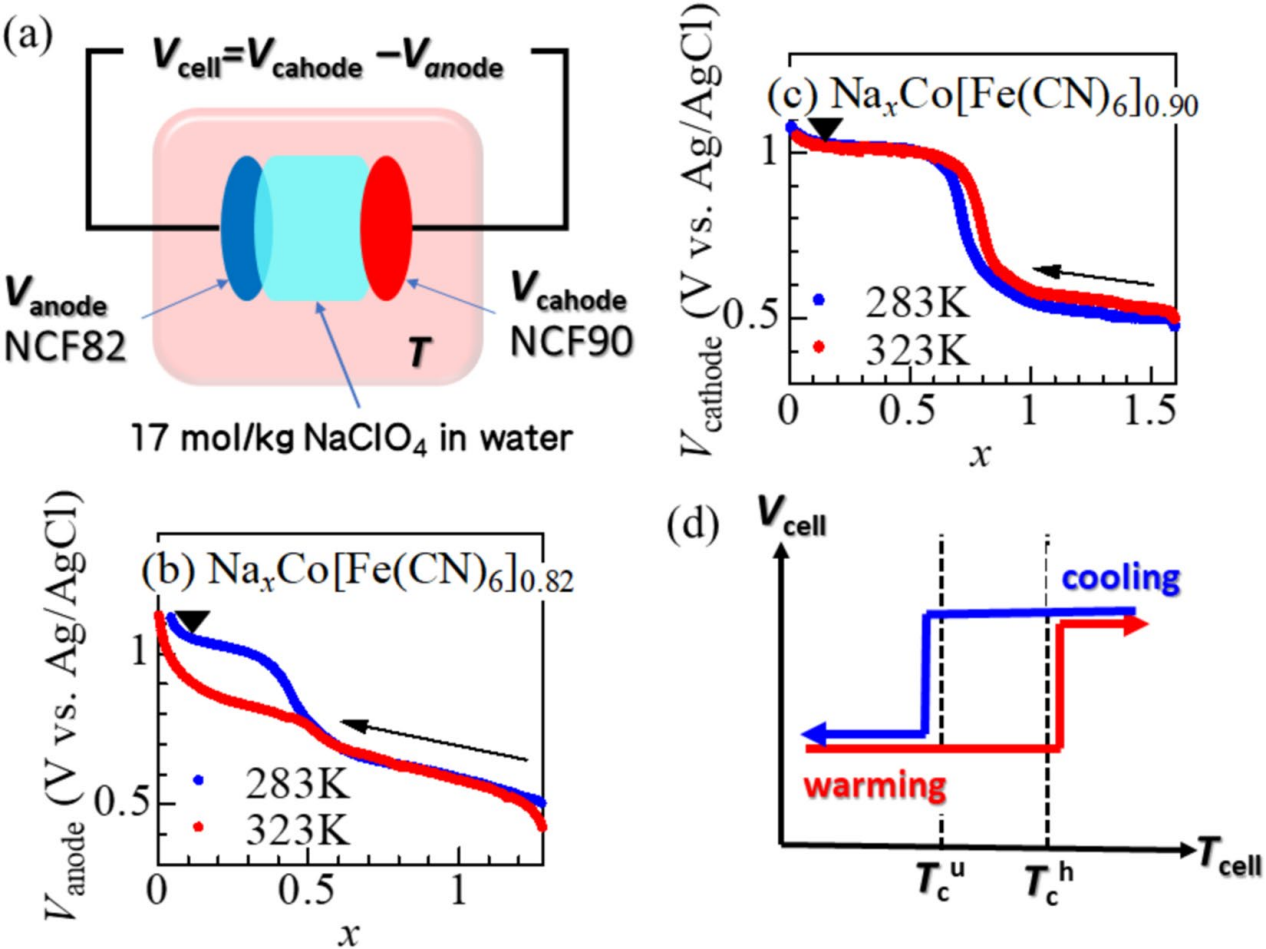
(**a**) Schematic illustration of tertiary battery. *V*_cell_, *V*_cathod_, *V*_anode_ are the cell voltage, redox potentials of cathode and anode materials, respectively. (**b**) Charge curves of the NCF82 film at various temperatures. The charge rate was 0.6 C. (**c**) Charge curves of the NCF90 film at various temperatures. The charge rate was 0.4 C. Closed triangles in (b) and (c) represent the actual *x* values of the pre-oxidized films for the tertiary battery. (**d**) Schematic illustration of *V*_cell_ against *T*_cell_. *T*_c_^u^ and *T*_c_^l^ are the upper and lower critical temperatures, respectively.

Figure 4(a) is schematic illustration of the tertiary battery. We fabricated NCF82/NCF90 tertiary battery, whose anode, cathode and electrolyte are the pre-oxidized NCF82 and NCF90 films and aqueous solutions containing 17 mol/kg NaClO_4_, respectively. Pre-oxidization of the films were performed at 1.01 V (*x* ~ 0.1) against Ag/AgCl in aqueous solutions containing 17 mol/kg NaClO_4_. The cell voltage (*V*_cell_) is expressed as *V*_cathode_ − *V*_anode_, where *V*_cathode_, *V*_anode_ are the redox potential of cathode and anode materials, respectively. As previously mentioned, *V*_anode_ of the NCF82 film [Fig. 4(b)] significantly drops by about 150 mV with increase in *T* from *T*_L_ to *T*_H_. This is in sharp contrast with *V*_cathode_ of the NCF90 film [Fig. 4(c)], which shows negligible temperature dependence over the entire range of *x*. Figure 4(d) shows schematic illustration of *V*_cell_ against *T*_cell_. In the warming process, the anode NCF82 film shows the LS–HS transition at *T*_cell_ = *T*_c_^u^. In the region of *T*_cell_ > *T*_c_^u^, *V*_cell_ (=*V*_cathode_ − *V*_anode_) becomes ~150 mV, since *V*_anode_ decreases by ~150 mV. In the cooling process, the anode NCF82 returns to the LS phase below *T*_cell_ = *T*_c_^l^. Then, *V*_cell_ returns to the initial value (=0 mV) in the region of *T*_cell_ < *T*_c_^l^.

**Figure 5:**
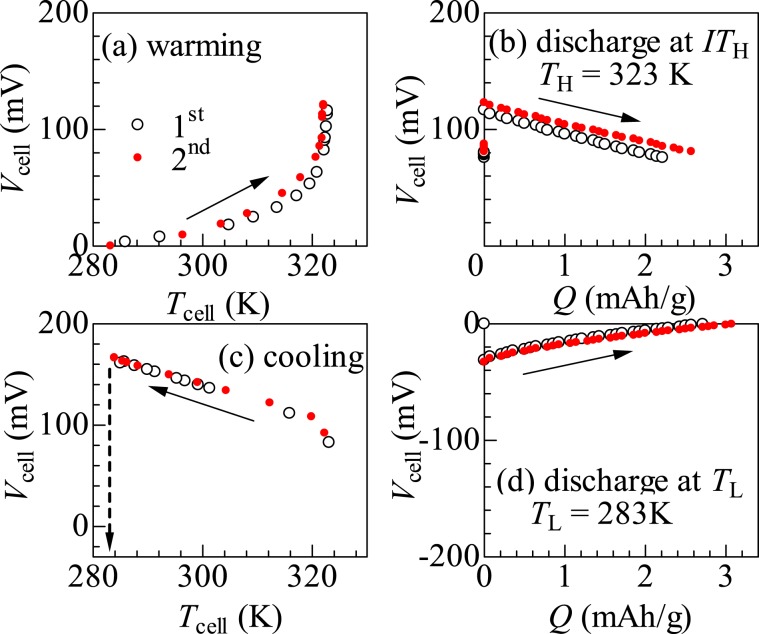
Thermal cycle of the NCF82/NCF90 cell: (**a**) Warming process from *T*_L_ (=283 K) to *T*_H_ (=323 K) in the open circuit condition, (**b**) discharge process at *T*_H_ at constant current at 0.7 C, (**c**) cooling process from *T*_H_ to *T*_L_ in the open circuit condition, and (**d**) discharge process at *T*_L_ at constant current at 0.7 C. *V*_cell_ and *T*_cell_ are the cell voltage and cell temperature, respectively. *Q* is the extracted charge per unit mass of NCF82. In the (**b**) discharge process at *T*_H_, lower limit of the voltage was set to 80 mV. In the (**d**) discharge process at *T*_L_, upper limit of the voltage was set to 0 mV. Open and closed symbols represent the data obtained in the first and second cycles, respectively. Broken arrow in (**c**) schematically represents the variation of *V*_cell_ during the change and discharge process at *T*_L_ at the rate of 0.7 C.

Figure 5 shows thermal cycle of the NCF82/NCF90 cell between *T*_L_ = 283 K (<*T*_c_^l^) and *T*_H_ = 323 K (>*T*_c_^u^). Open and closed symbols represent the data obtained in the first and second cycles, respectively. In the (a) warming process, *V*_cell_ gradually increases with *T*_cell_ and eventually divergently increases around 320 K. Such a divergently increases in *V*_cell_ is ascribed to the LS–HS transition of the NCF82 film at *T*_c_^u^ = 325 K (at *x* ~ 0.1). We note that any redox reaction nor Na^+^ transportation takes place in the warming process, because the process is performed in the open circuit condition. The measurement above *T*_cell_ = 323 K is difficult due to sample detonation. At *T*_H_, the cell shows a huge voltage (=120 mV). In the (b) discharge process at *T*_H_, *V*_cell_ linearly decreases with the extracted charge (*Q*) per unit mass of NCF82. The final extracted charge (*Q*_NCF82_) from NCF82 is 2.4 mAh/g, which is 2.3% of the discharge capacity of NCF82. More specifically, the Na concentration (*x*) of NCF82 (NCF90) decreases (increases) by 0.03 (0.004) in the discharge process at *T*_H_. The electric work (*W*_H_ = 2.6 meV/NCF82) at *T*_H_ is roughly evaluated by integration of *V*_cell_ over *Q*. Here, let us explain how the *x* value in the NCF82 anode changes during an ideal thermal cycle. We note that the NCF90 cathode works as Na storage because the area of the NCF90 cathode is much larger than the area of NCF82. The *x* value of the pre-oxidized NCF82 is 0.1. In the (a) warming process in the open circuit condition, *x* remains at the initial value (~0.1). The warming process causes the thermally-induced phase transition from the LS phase to the HS phase. In the (b) discharge process at *T*_H_, *x* decreases from 0.1 to 0.07. In the (c) cooling process in the open circuit condition, *x* remains at 0.07. The cooling process causes the thermally-induced phase transition from the HS phase to the LS phase. In the (d) discharge process at *T*_L_, *x* increases from 0.07 to 0.1.

In the (c) cooling process, *V*_cell_ gradually increases even if *T*_cell_ crosses *T*_c_^u^ = 305 K (at *x* ~ 0.1). This unexpected behaver is probably ascribed to the residual HS phase, which is discernible in the XRD pattern around the (200) reflection as a tail structure (Fig. S5). We note that the redox potential of the HS phase is lower than that of the LS phase. This means that the residual HS phase governs the redox process. We found that the residual HS phase can be eliminated by the charge and discharge processes at *T*_L_ (Fig. S6). *V*_cell_ returns to the initial value (~0 mV) after the charge and discharge processes at *T*_L_. In the (d) discharge process, *Q*_NCF82_ is 3.0 mAh/g, which is 2.9% of the discharge capacity of NCF82. In this process, *x* of NCF82 (NCF90) returns to the initial value (~0.1). The electric work (*W*_L_) at *T*_L_ is 0.7 meV/NCF82.

The unexpected phase mixing at *T*_L_ is probably ascribed the distribution of the chemical composition among the crystals in the film. In Co-PBA, *T*_c_ of the LS–HS transition is significantly sensitive to the *y* value (Fig. S1). One percent increase in *y* increases *T*_c_ by ~ 10 K. It is interesting that the residual HS phase is eliminated by the charge and discharge processes at *T*_L_. In the charge process of the tertiary battery, the anode film is reduced to the initial as-grown state, i.e., Na_4*y*−2_Co^II^[Fe^II^(CN)_6_]_*y*_ (*y* ~ 0.82). In the discharge process of the tertiary battery, the anode film is partly oxidized. In this process, high-*y* particles (in the HS phase) with lower-*V* are selectively oxidized to Co^II^_3–3*y*_Co^III^_3**y**−2_[Fe^III^(CN)_6_]_*y*_ and become electrochemically inert. Then, the redox potential is dominated by the low-*y* particle in the LS phase after the charge and discharge process at *T*_L_.

## Thermal Efficiency

Let us roughly evaluate the thermal efficiency (*η* = *W*/*Q*, where *W* and *Q* are the output work and input thermal energy) of the NCF82/NCF90 cell. In the initial cycle, *W* = 3.3 meV/NCF82. The input thermal energy is *C* (*T*_H_ − *T*_L_) + *H*, where *C* and *H* are the sum of the specific heats of the anode and cathode materials and latent heat for the phase transition. We neglect the specific heat of the electrolyte, since the amount of electrolyte can be minimized in a battery. We used calculated *C* (=4.16 meV/K) of ideal Na_2_Co[Fe(CN)_6_] in the high-temperature limit (the Dulong-Petit law). *H* (18.3 meV) of the NFC82 film at *x* ~ 0.1 was evaluated by differential scanning calorimetry (DSC). Then, *Q* is roughly evaluated to be 351.1 meV. Thus, we obtained *η* = 0.9%, which is 11% of the Carnot efficiency (*η*_carnot_ = 1 − *T*_L_/*T*_H_) between *T*_L_ (=286 K) and *T*_H_ (=313 K).

## Summary

We demonstrated that the usage of the LS–HS transition of Co-PBA qualitatively improved performance of the tertiary battery. *V*_cell_ of the NCF82/NCF90 cell steeply increase from 0 mV to ~120 mV around 320 K. Our observation indicates that the tertiary battery with use of phase transition is a promising independent power supply for the IT/IoT devices. However, the demand for the chemical and physical uniformity in the electrode material is much severer in the tertiary battery with use of the phase transition than that in the conventional secondary battery. To realize the chemical and physical uniformity, improvement of the sample synthesis is under progress.

## Method

### Fabrication and characterization of Co-PBA films

Thin films of Na_*x*_Co[Fe(CN)_6_]_0.82_3.5H_2_O (NCF82) and Na_*x*_Co[Fe(CN)_6_]_0.9_2.9H_2_O (NCF90) were synthesized by electrochemical deposition on an indium tin oxide (ITO) transparent electrode under potentiostatic conditions at −0.45 V vs a standard Ag/AgCl electrode. The electrolytes were aqueous solution containing 0.8 mmol/L (0.8 mmol/L) K_3_[Fe^III^(CN)_6_], 0.5 mmol/L (0.5 mmol/L) Co^II^(NO_3_)_2_, and 0.5 mol/L (5.0 mol/L) Na(NO_3_) for the NCF82 (NCF90^19^) film. In this process, the reduction reaction of [Fe^3+^(CN)_6_]^3− + ^e^−^ ➔ [Fe^2+^(CN)_6_]^4−^ triggers the deposition of Co-PBA. Therefore, Fe and Co in the as-grown films are divalent. The deposition time is ~30 minutes. The obtained film was transparent (Fig. S7) with a thickness of ~500 nm. The chemical compositions of the films were determined using the inductively coupled plasma (ICP) method and CHN organic elemental analysis. The surface scanning electron microscopy (SEM) images of the films reveals that the films consist of cubic shape crystals of several hundred nm (Fig. S8). The crystal structure of the as-grown NCF82 (NCF90) film was trigonal with *a*_H_ = 7.352(8) Å and *c*_H_ = 17.519(22) Å [*a*_H_ = 7.408(9) Å and *c*_H_ = 17.476(24) Å] (Fig. S9). The NCF82 film transfers to the fcc structure below *x* = 0.96 (Fig. S2).

### Electrochemical measurement

The charge/discharge curves of the Co-PBA films were measured with a potentiostat (HokutoDENKO, HJ1001SD8) using a three-pole beaker-type cell. The working, referential, and counter electrodes were the film, a standard Ag/AgCl electrode, and Pt, respectively. The electrolyte is aqueous solution containing 17 mol/kg NaClO_4_. The charge/discharge rate was about 0.4–0.6 C. The upper and lower limits of the redox potential were 0.20 and 1.1 V vs. Ag/AgCl, respectively. The mass of each film was evaluated using thickness, area, and density. We confirmed that the actual densities of the NCF82 and NCF90 films were 0.98 and 0.58 of the ideal density, respectively. The discharge capacity (=104 mAh/g) of the NCF82 film was closed to the ideal value (=105 mAh/g) for the Na intercalation from *x* = 0.0 to 1.28. The discharge capacity (=140 mAh/g) of the NCF90 film was closed to the ideal value (=127 mAh/g) for the Na intercalation from *x* = 0.0 to 1.60.

### *Ex situ* XRD measurement

For the *ex situ* X-ray powder diffraction (XRD), the magnitude of *x* of the NCF82 film was controlled by the charge/discharge process in a beaker-type cell. The working, referential, and counter electrodes were the film, a standard Ag/AgCl electrode, and Pt, respectively. The electrolyte is aqueous solution containing 17 mol/kg NaClO_4_. The charge/discharge rate was about 1.0 C. The active areas of the films were about 1.0 cm^2^. The cut-off voltage was from 0.2 to 1.1 V. The magnitude of *x* was evaluated from the extracted charge under the assumption that Na_1.28_Co[Fe(CN)_6_]_0.82_ is in the discharged state and Na_0.00_Co[Fe(CN)_6_]_0.82_ is in the fully-charged state. The relative error of *x* is ~ 0.05.

The XRD patterns of the *x*-controlled NCF82 film were measurand in the θ−2θ geometry with use of with an X-ray diffractometer (Rigaku, RINT2000PC). The film was attached at the cold heat of a cryostat, whose temperature was controlled liquid N_2_ and electric heater. The X-ray source was the Cu Kα line at 40 kV and 40 mA. A Si monochromator was used to reduce the scattering by white X-ray. Typical measurement time of XRD pattern is 10 minute/degree. We note that Co-PBA will deteriorate if left at high temperature (>350 K) for a long time. In order to shorten the measurement time, only the *T*-dependence of the (002) reflection in the cubic cell was investigated. The lattice constant (*a*) was calculated with use of Bragg’s law.

### Thermal cycle measurement of the battery

We fabricated a two-pole beaker-type tertiary battery, whose anode, cathode and electrolyte are the pre-oxidized NCF82 and NCF90 films and aqueous solutions containing 17 mol/kg NaClO_4_, respectively. Pre-oxidization of the films were performed at 1.01 V against Ag/AgCl in aqueous solutions containing 17 mol/kg NaClO_4_. The *x* value is ~ 0.1 for both the NCF82 and NCF90 films. In the present experiment, we concentrated our attention on the voltage jump of NCF82 (anode) at the phase transition, and hence, the NCF90 (cathode) was regarded as the fixed point of the redox potential. For this purpose, the film area (2.0 cm^2^) of cathode is much larger than that (0.25 cm^2^) of anode to minimize the variation of *x*, and hence the redox potential, of cathode.

The thermal cycle measurement consists of four processes: (a) warming process from *T*_L_ (=283 K) to *T*_H_ (=323 K), (b) discharge process at *T*_H_, (c) cooling process from *T*_H_ to *T*_L_, and (d) discharge process at *T*_L_. In the (a) warming process, device temperature (*T*_cell_) was slowly increased from *T*_L_ to *T*_H_ in the open circuit condition. *T*_cell_ was monitored by a Pt resistance thermometer in the electrolyte. At (b) *T*_H_, the thermally-charged cell was discharged at 0.7 C. The discharge rate was defined by the NCF82 film. In the (c) cooling process, *T*_cell_ was slowly decreased from *T*_H_ to *T*_L_ in the open circuit condition. In order to obtain the purely LS phase without residual HS phase, charge and discharge processes at *T*_L_ were performed at the rate was 0.7 C. The upper and lower limits of voltage was 0.70 and −0.04 V, respectively. The lower limit of voltage (=−0.04 V) was determined so that *x* becomes the same value after the (c) discharge process at *T*_H_. At (d) *T*_L_, the thermally-charged cell was discharged at 0.17 C.

## Supplementary information

Supplementary Information.
