# Supplementary material for: Energy harvesting thermocell with use of phase transition
Source: Sci Rep. 2020 Feb 4;10:1813. doi: 10.1038/s41598-020-58695-z (PMC7000752; doi:10.1038/s41598-020-58695-z)
Supplement: Supplementary file 1 — Supplementary Information. [file 41598_2020_58695_MOESM1_ESM.pdf]

Supporting information

Energy harvesting thermocell with use of phase transition

Takayuki Shibata[1*], Hiroki Iwaizumi[2], Yuya Fukuzumi[2], and Yutaka Moritomo[2-4*]

[1]National Institute of Technology, Gunma College, Maebashi, Gunma 371-8530, Japan

[2]Graduate School of Pure and Applied Sciences, University of Tsukuba, Tsukuba 305-8571, Japan

[3]Faculty of Pure and Applied Sciences, University of Tsukuba, Tsukuba 305-8571, Japan

[4]Tsukuba Research Center for Energy Materials Science (TREMS), University of Tsukuba, Tsukuba 305-8571, Japan

Contact information:

Yutaka Moritomo

Graduate School of Pure and Applied Sciences

Faculty of Pure and Applied Sciences, and

Tsukuba Research Center for Energy Materials Science (TREMS)

Univ. of Tsukuba, Tennodai 1-1-1, Tsukuba 305-8571, Japan

E-mail: moritomo.yutaka.gf@u.tsukuba.ac.jp

Takayuki Shibata

National Institute of Technology

Gunma College, Maebashi, Gunma 371-8530, Japan

E-mail: shibata@nat.gunma-ct.ac.jp

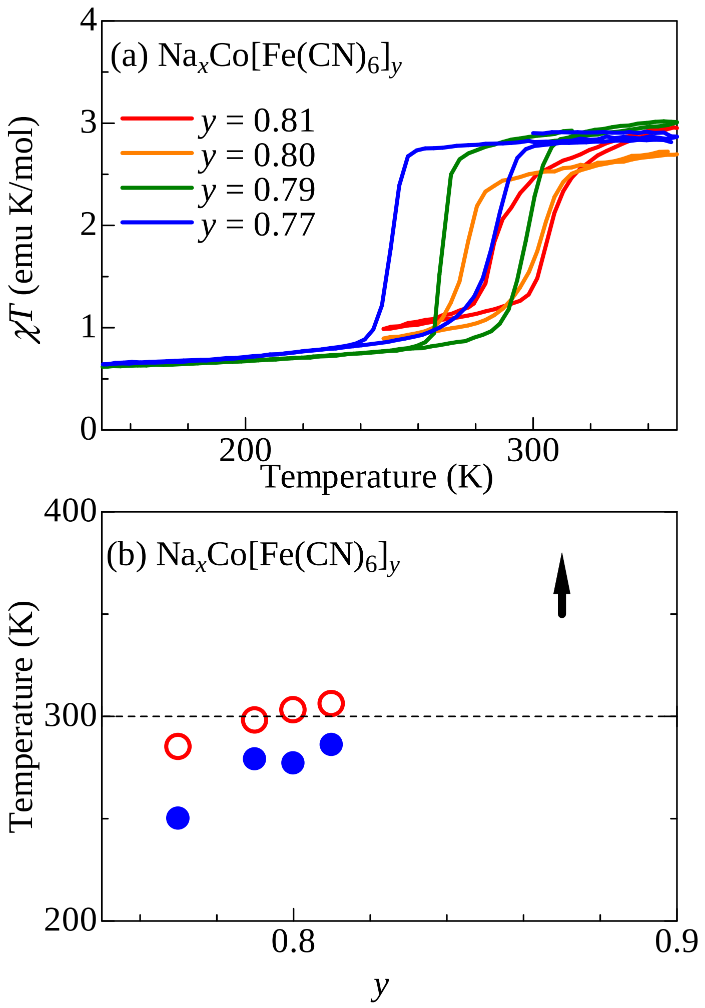

Figure S1. (a) Temperature (T) dependence of Magnetic susceptibly (χ) of $Na_xCo[Fe(CN)_6]_y$ powder against Fe concentration (y). Sudden increase of χT is ascribed to the LS – HT phase transition. (b) Upper (T_c^u) and lower (T_c^l) critical against y. T_c^u (T_c^l) are defined by the temperature corresponding to the midpoint of χT between the high temperature and low temperature sides in the warming (cooling) run. An upper arrow in (b) means that no phase transition is observed below 350 K[1].

References

1. Shimamoto, N., Ohkoshi, S., Sato, O. & Hashimoto, K. Control of charge-transfer-induced spin transition temperature on cobalt-iron Prussian blue analogues. *Inorg. Chem.*, 41, 678-684 (2002)

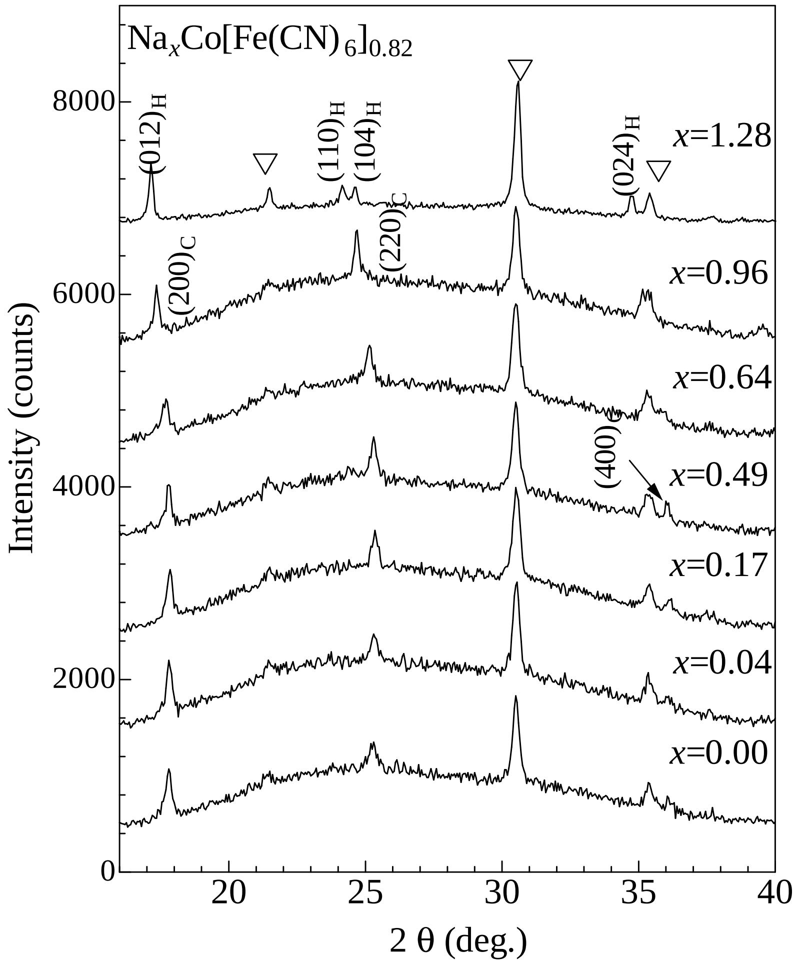

Figure S2. X-ray diffraction (XRD) patterns of the NCF82 films against x at 300 K. The X-ray source was the CuKα line. At x = 1.28, NCF82 shows trigonal ($R\bar{3}m$; Z = 3) structure. Numbers in parentheses represent the index in the trigonal cell hexagonal setting. Below x = 0.96, NCF82 shows face-centered cubic (fcc) ($Fm\bar{3}m$; Z = 4) structure. Numbers in parentheses represent the index in the cubic cell. Triangles represent diffraction peaks due to ITO.

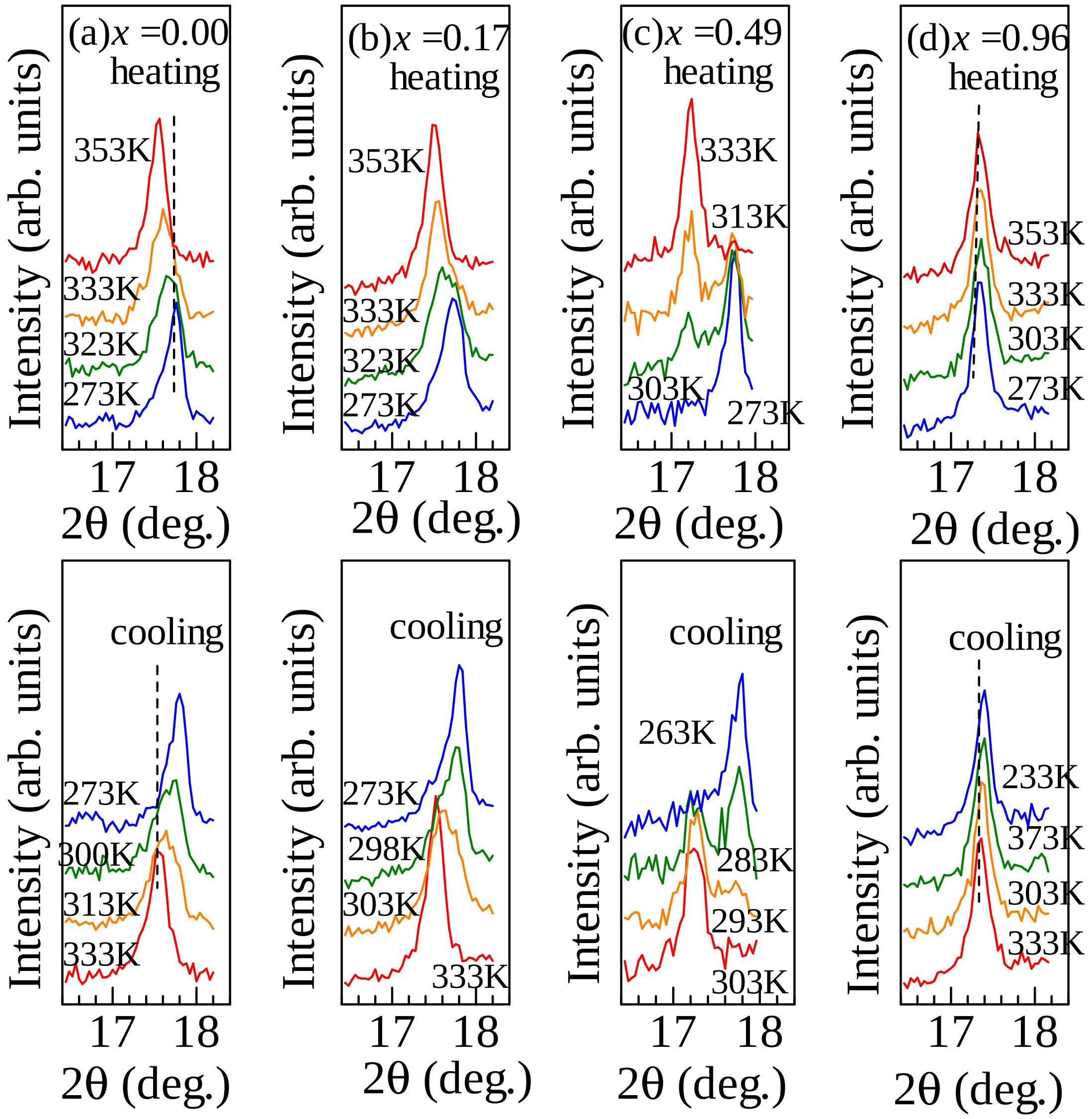

Figure S3. Temperature dependence of the XRD pattern of the NCF82 film around the (200) reflection: (a) x = 0.00, (b) 0.17, (c) 0.49, and (d) 0.96. Upper and lower panel represent for the heating and cooling runs, respectively. The X-ray source was the CuKα line.

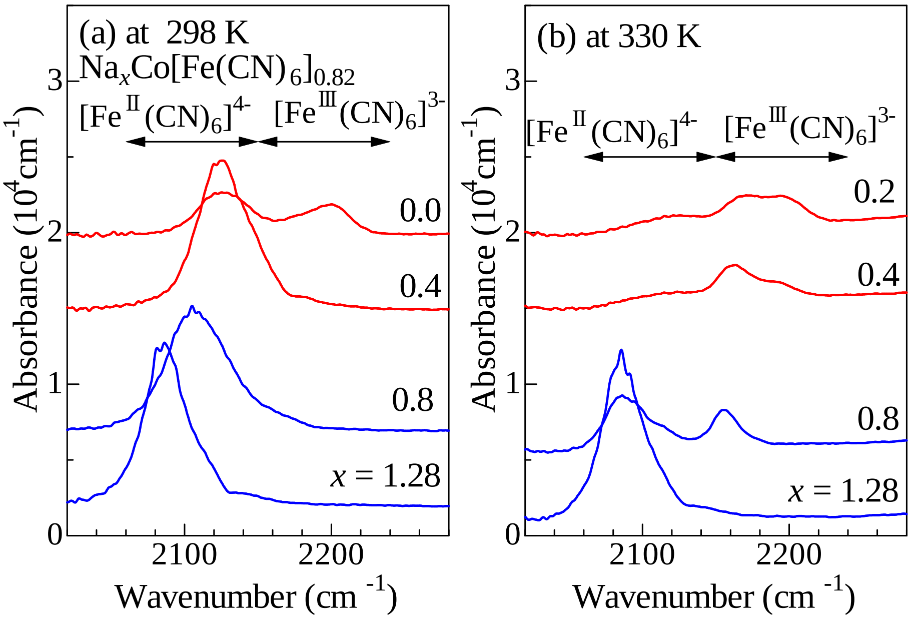

Figure S4. Infrared (IR) absorption spectra of the NCF82 films against *x* at 300 K. Blue (red) curve represents the spectra in the lower-lying (higher-lying) plateau. Horizontal arrows indicate the spectral regions where CN stretching vibrational mode appears in the $[Fe^{III}(CN)_6]^{3-}$/$[Fe^{II}(CN)_6]^{4-}$ units.

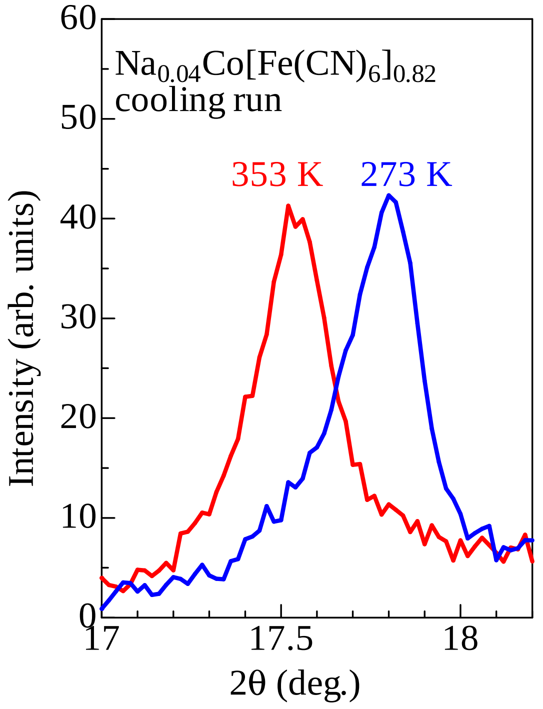

Figure S5. XRD pattern around the (200) reflection of NCF82 film (x =0.04) at 353 K (> T_c^u = 325 K) and 273 K (< T_c^l =320 K). AT 273 K, a tail structure in the lower-angle side is discernible, suggesting residual LS phase.

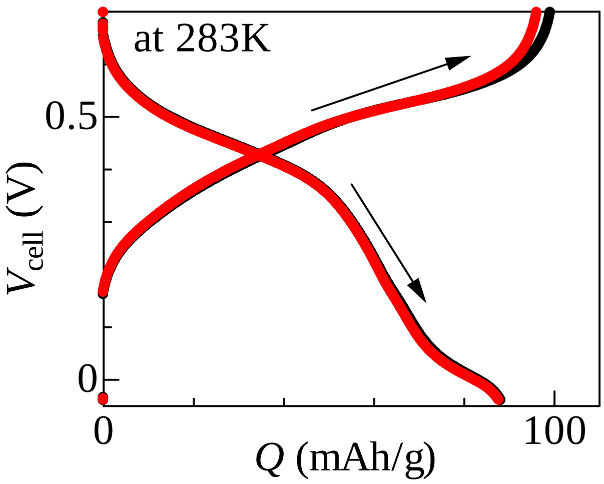

Figure S6. Change and discharge curve of NCF82/NCF90 cell at T_L (= 283 K). The rate was 0.7 C. The upper and lower limits of voltage was 0.70 and - 0.04 V, respectively. The lower limit of voltage (= - 0.04 V) was determined so that x becomes the same value after the (c) discharge process at T_H (= 323 K). Q is the extracted charge per unit mass of NCF82.

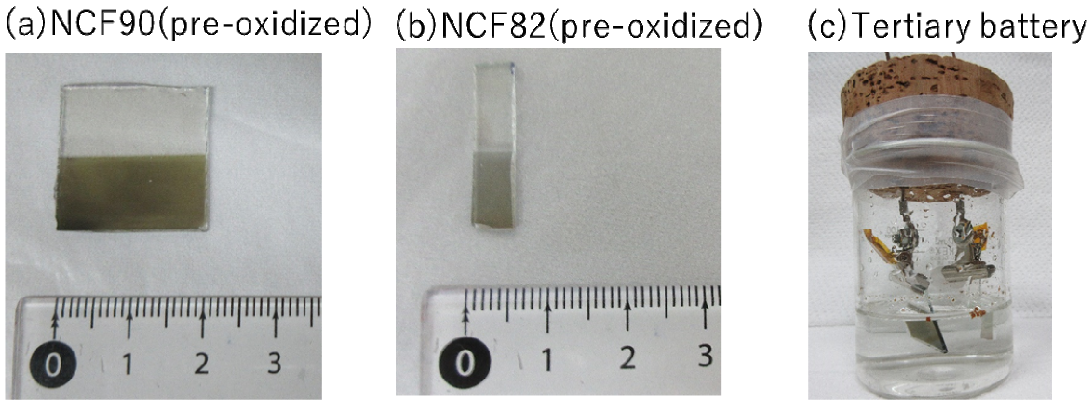

Figure S7. Pictures of (a) pre-oxidized NCF82 (x ~ 0.1), (b) pre-oxidized NCF90 (x ~ 0.1) films, and (c) beaker-type tertiary battery.

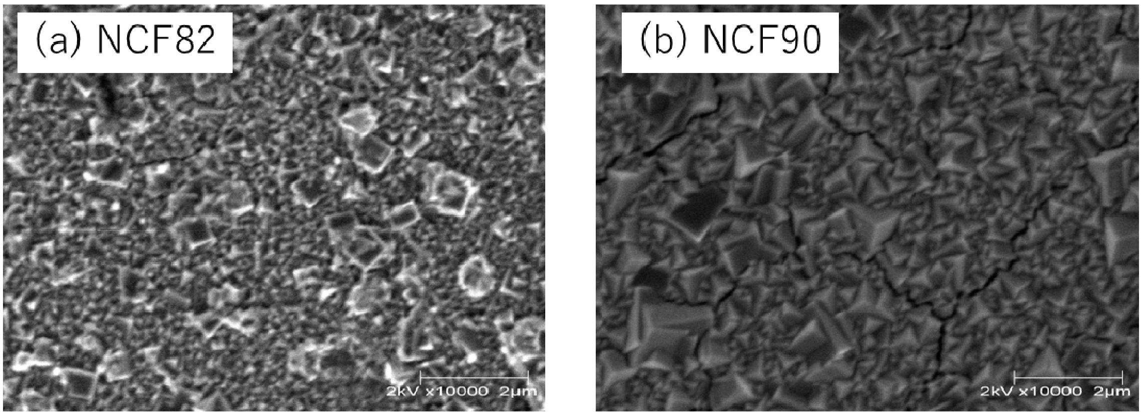

Figure S8. SEM image of (a) NCF82 and (b) NCF90 films. The acceleration voltage was 2 kV.

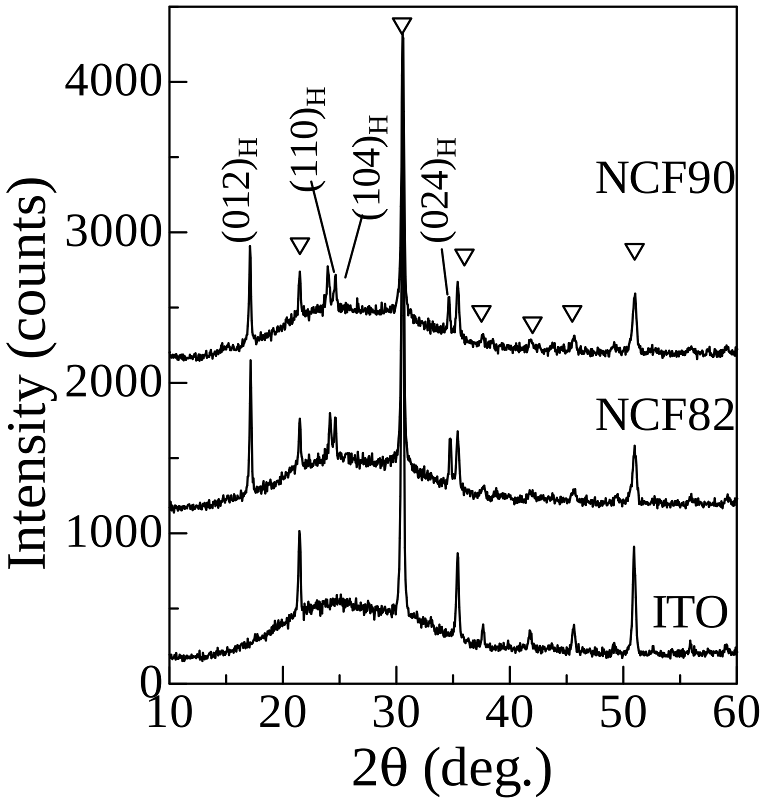

Figure S9. X-ray diffraction pattern of as-grown NCF90 and NCF82 films at 300 K. The X-ray source was the CuKα line. Numbers in parentheses represent the index with the trigonal ($R\overline{3}m$; $Z = 3$) cell in the hexagonal setting. The bottom pattern is for the ITO substrate without the PBA lime. Triangles represent the diffraction peaks due to ITO.
